# Laparoscopic Conversion to Open Cholecystectomy: Is Incidental Gallbladder Cancer Any Different?

**DOI:** 10.7759/cureus.62187

**Published:** 2024-06-11

**Authors:** Trevor S Silva, Matthew Firek, Paul Albini, David Caba Molina

**Affiliations:** 1 Hepatobiliary Surgery, Portland Providence Medical Center, Portland, USA; 2 Comparative Effectiveness and Clinical Outcomes Research Center (CECORC), Riverside University Health System Medical Center, Moreno Valley, USA; 3 General Surgery, Riverside University Health System Medical Center, Moreno Valley, USA; 4 Surgical Oncology, Loma Linda University Health, Loma Linda, USA

**Keywords:** conversion to open cholecystectomy, laparoscopic cholecystectomy, incidental gallbladder cancer, gallbladder cancer, biliary cancer

## Abstract

Background: A majority of gallbladder cancers present incidentally. Operative risk factors and outcomes for laparoscopic converted to open cholecystectomy in incidental gallbladder cancer are not well characterized.

Methods: Patients with incidental gallbladder cancer and acute cholecystitis undergoing laparoscopic cholecystectomy and conversion to open cholecystectomy in the National Surgical Quality Improvement Program (NSQIP) database of the American College of Surgeons (ACS) from 2010-2019 were reviewed. The primary endpoint was risk factors for conversion to open cholecystectomy in incidental gallbladder cancer. Chi-squared test or Fisher’s exact test was used for categorical variables. Continuous variables were compared using the Mann-Whitney U test.

Results: A total of 5,789 patients undergoing laparoscopic cholecystectomy were identified, of which, 50 (0.9%) had incidental gallbladder cancer. For incidental gallbladder cancer patients, there were no differences in preoperative profile and risk factors between laparoscopic and converted to open cholecystectomy groups. Incidental carcinoma patients undergoing conversion to open cholecystectomy had lower preoperative sodium levels than the laparoscopic cholecystectomy group (P=0.007).

Hospital length of stay (days) was longer for those with a conversion to open cholecystectomy for incidental carcinoma compared to non-conversion, 14 (10.8, 18.8) vs 2 (0.3, 5) (P=0.004). The patients converted to open cholecystectomy also had higher rates of postoperative sepsis (50% vs 0%, P<0.001) and reoperation than the laparoscopic cohort (50% vs 2.2%, P<0.001). Readmission and mortality rates, among other complications, were not significantly different between both surgical approaches in incidental gallbladder cancer patients.

Conclusions: Patients with conversion to open cholecystectomy had worse outcomes including longer hospital stays and higher rates of sepsis and reoperation. It remains difficult to predict which incidental gallbladder patients will require a conversion to open surgery. Further study examining whether more complicated recovery results in worse oncologic outcomes is warranted.

## Introduction

Gallbladder cancer is a rare form of carcinoma, with a prevalence of fewer than 2/100,000 individuals worldwide [1] and 1.4/100,000 individuals in the United States [2]. Risk factors for gallbladder carcinoma include gallstones, female sex, large polyps, obesity, and belonging to certain populations (Native Americans, Bolivians, Nepalese, etc.) [2,3]. Approximately 50% of gallbladder cancers are discovered incidentally at cholecystectomy [4]. However, the prevalence of incidental gallbladder cancer (iGBC) on surgical pathology for a patient undergoing cholecystectomy for a benign indication is reported to be 0.7% [5].

Overall survival rates for gallbladder cancer are <5% at five years [3]. Whether or not gallbladder cancer discovered incidentally has a worse outcome is not well characterized [5]. The standard of care for gallbladder cancer surgery remains an open approach, whether suspected intra-operatively or taken back for a re-resection [6]. However, recent studies have shown no difference in oncologic outcomes between the open and laparoscopic approaches [7,8].

Risk factors for conversion to open cholecystectomy (COC) for benign disease have been extensively studied. These variables include male sex, acute cholecystitis, high BMI, advanced age, and specific laboratory results such as elevated alkaline phosphatase [9-11]. iGBC itself has been recognized as a risk factor for COC [4,12,13].

As COC may prolong recovery, the implications may be greater in patients with a malignant diagnosis as additional therapy may be warranted. Delays in adjuvant therapy may lead to worse long-term outcomes. It remains unclear whether the same risk factors for COC in benign disease apply to incidental carcinoma. The primary aim of this study was to identify risk factors for COC in cases of iGBC. Secondary aims included surgical outcome differences between laparoscopic cholecystectomy (LC) and COC for iGBC. 

This article was previously presented as a meeting abstract at the annual Americas Hepato-Pancreato-Biliary Association (AHPBA) conference in Miami, Florida, August 2-5, 2021, and the 15th International Hepato-Pancreato-Biliary Association (IHPBA) World Congress in New York City, New York, March 30-April 2, 2022. 

## Materials and methods

This study is a review of the American College of Surgeons' National Surgical Quality Improvement Program (ACS-NSQIP) database. This database is a multi-institutional collaboration that serves the purpose of measuring and improving outcomes of surgical care. Today, almost 700 hospitals participate in the database, providing preoperative, intraoperative, and postoperative variables. These variables include, but are not limited to, comorbidities, laboratory values, complications, and mortalities. The database tracks surgical outcomes and complications for 30 days postoperatively. The data for each participant makes up a participant user data file (PUF). Diagnoses and surgical procedures are designated in the database using the International Classification of Diseases (ICD) and Current Procedural Terminology (CPT) codes, respectively.

PUF from 2010-2019 were selected using ICD 9 and 10 codes for acute cholecystitis (575.0 and K81.0, respectively) and gallbladder cancer (156.0 and C23, respectively). Acute cholecystitis was chosen as a control due to its prevalence as a benign indication for surgery and the diagnosis being an independent risk factor for conversion to open cholecystectomy [14]. Six CPT codes (2018) for cholecystectomy were chosen. Three for LC (47562, 47563, 47564) and three for open cholecystectomy (47600, 47605, 47610). The diagnoses from the NSQIP database are postoperative, therefore the gallbladder cancer diagnoses were treated as incidental. 

Laparoscopic COC was designated as the surgical intervention for every PUF that included CPT codes for both LC and open cholecystectomy, as previously described [4]. NSQIP variables reviewed were broadly grouped into: demographics/comorbidities, preoperative labs, and postoperative course/complications. Our primary outcome was COC in iGBC cases, and we investigated risk factors for conversion. Secondary outcomes were postoperative complications, including 30-day mortality, surgical site infections, sepsis, and reoperation.

Exclusion criteria included: a history of disseminated cancer, chemotherapy within 30 days of surgery, and radiotherapy within 90 days of surgery. The statistical analysis was completed using R version 3.6.0 (R Foundation for Statistical Computing, Vienna, Austria). Statistical analyses comprised Chi-squared and Fisher’s exact tests for categorical variables. Continuous variables in a normal distribution were compared with t-tests, and non-normal variables were compared with quantiles and the Mann-Whitney U test. A p-value of less than 0.05 was designated as statistically significant. This study was exempted from review by the Riverside University Health System Institutional Review Board.

## Results

**Figure 1:**
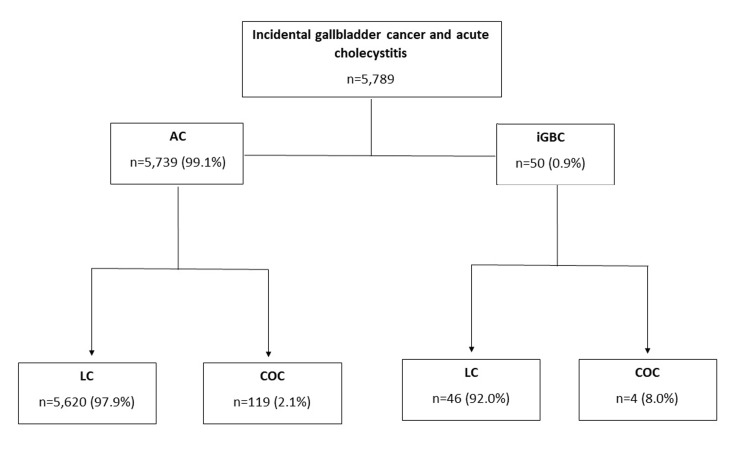
Incidental gallbladder patient cohorts and their rates of conversion to open cholecystectomy AC: acute cholecystitis; iGBC: incidental gallbladder cancer; LC: laparoscopic cholecystectomy; COC: converted to open cholecystectomy

In this review of 5,789 patients undergoing LC and COC, a 0.9% (n=50) prevalence of iGBC was identified. Four (8.0%) of the iGBC patients underwent a COC, compared to 119 (2.1%) (P=0.02) in the acute cholecystitis group. Figure 1 gives a schematic breakdown of the patient cohorts. There were no significant differences in preoperative profiles between the LC and COC cases in the iGBC cohort. A complete description of the preoperative iGBC cohort characteristics is given in Table 1. Significantly lower preoperative median (range) sodium levels (133 (132,135) vs 139 (137,141) (P=0.007)) were seen in the COC cohort. Table 2 gives a review of preoperative laboratory values in both cohorts. There was no difference in time from presentation to operation between the two groups. American Society of Anesthesiologists (ASA) status and Wound Class were omitted from the analysis as the iGBC COC group (n=4) could not be divided.

**Table 1:** Preoperative characteristics for incidental gallbladder cancer patients BMI: body mass index; COPD: chronic obstructive pulmonary disease

Characteristics	No Conversion (N=46), n (%)	Conversion (N=4), n (%)	P-value
Insulin-dependent diabetes	4 (8.7)	0 (0)	0.70
Non-insulin-dependent diabetes	9 (19.6)	0 (0)	0.70
Male sex	11 (23.9)	1 (25)	>0.99
Smoking history	4 (8.7)	0 (0)	>0.99
Emergency surgery	3 (6.5)	0 (0)	0.60
BMI (kg/m^2^), Median (range)	30.0 (24.0, 36.0)	24.2 (23.6, 24.8)	0.06
COPD	3 (6.5)	1 (25)	0.49
Weight loss	1 (2.2)	0 (0)	0.25
Pre-operative sepsis	2 (4.3)	0 (0)	>0.99
Congestive heart failure	1 (2.2)	0 (0)	>0.99
Hypertension	30 (65.2)	4 (100)	0.29
Bleeding disorder	4 (8.7)	1 (25)	0.35

**Table 2:** Preoperative laboratory values for incidental gallbladder cancer patients IQR: interquartile range; BUN: blood urea nitrogen; WBC: white blood cell; PTT: partial thromboplastin time; INR: international normalized ratio

LAboratory tests	No Conversion, median (IQR)	Conversion, median (IQR)	P-value
Sodium, mmol/L	139 (137,141)	133 (132,135)	0.007
BUN, mg/dL	16 (12.8,20.72)	13 (13,13.92)	0.46
Creatinine, mg/dL	0.9 (0.7,1.15)	0.77 (0.73,0.84)	0.48
Albumin g/dL	4.05 (3.48,4.5)	4.5 (3.7,4.55)	0.75
Total bilirubin, mg/dL	0.6 (0.5,0.9)	0.41 (0.35,0.5)	0.38
Alkaline phosphatase, units/L	82 (67,102)	65 (59.5,240.5)	0.67
WBC count, x10^3^/mm^3^	7.3 (5.65,10.2)	9.35 (8.03,10.05)	0.75
Hematocrit, %	39.6 (34.7,41.05)	36.9 (36.67,37.25)	0.56
Platelet count, x10^3^	233 (192,289.75)	222 (206.75,257)	0.99
PTT, second	30.7 (30,32.4)	30.3 (30.3,30.3)	0.77
INR	1 (0.99,1.17)	1.22 (1.13,1.31)	0.18

Hospital length of stay (mean days) was longer for those with COC compared to LC, 14 days (10.8, 18.8) vs two days (0.3, 5) (P=0.004). COC also had higher rates of postoperative sepsis (50% vs 0%, P<0.001) and reoperation than LC (50% vs 2.2%, P<0.001). Readmission and 30-day mortality rates, among other complications, were not significantly different between COC and LC. Surgical site infections were omitted from the analysis as the iGBC COC group (n=4) could not be divided. See Table 3 for a complete list of postoperative complications.

**Table 3:** Postoperative complications for incidental gallbladder cancer

Complications	No Conversion (N=46), n (%)	Conversion (N=4), n (%)	P-value
Death	1 (2.2)	0 (0)	>0.99
Readmission within 30 days	9 (19.6)	2 (50)	0.16
Pneumonia	1 (2.2)	1 (25)	0.16
Renal Insufficiency	1 (2.2)	0 (0)	>0.99
Urinary tract infection	1 (2.2)	0 (0)	>0.99
Cardiac arrest	1 (2.2)	0 (0)	>0.99
Myocardial infarction	1 (2.2)	0 (0)	>0.99
Bleeding requiring transfusion	1 (2.2)	1 (25)	0.16
Sepsis	0 (0)	2 (50)	<0.001
Reoperation	1 (2.2)	2 (50)	<0.001

## Discussion

iGBC was a rare entity (0.9%) in this cohort of over 5,000 patients undergoing cholecystectomy. When compared to surgery for a benign diagnosis, incidental carcinoma had a higher rate of COC. Among patients with iGBC, those requiring conversion to open surgery had longer lengths of stay, as well as higher rates of postoperative sepsis and reoperation.

The higher incidence of COC in the iGBC cohort in this study corroborates with data from a prior review. Dorobisz et al. also found a greater rate of COC for iGBC compared to benign cases [15]. A possible confounder in our study is open cholecystectomy being the current approach to gallbladder cancer, although, without intraoperative reports, one cannot assume that the suspicion of carcinoma exclusively led to conversion.

The impact of BMI on the rate of COC for a benign surgical indication has been examined with mixed results. An 11.7% conversion rate in obese (BMI ≥ 30.0 kg/m²) compared to 6.1% in non-obese patients (P=0.0003) was noted in one study of over 1,500 surgeries [16]. However, two studies did not find a difference in conversion rates among patients stratified into BMI categories ranging from 18.5 to ≥ 40 (P = 0.4) [17,18]. Similarly, our study did not note an association between elevated BMI and COC in the iGBC cohort. It should be noted that the median BMI of both iGBC LC and COC groups in this study was at or below a BMI of 30.

Male gender [19,20], diabetes [21], and elevated preoperative alkaline phosphatase [22] have been found to be associated with conversion in benign gallbladder disease. In contrast, our study did not identify in iGBC cases a significant association between COC and the male sex or diabetes. The only significant difference in preoperative laboratory studies was an association between lower median sodium and COC. The previously reported preoperative risk factors for COC in benign gallbladder disease were not found to be associated with COC for iGBC. The authors hypothesize that a chronic inflammatory state, which may lead to the development of carcinoma, could be the driving force for the negative outcomes noted in this study. The chronic inflammatory reaction may portend to a more difficult operation, leading to open surgery. Further investigation is warranted into identifying and differentiating the risk factors for COC in benign and iGBC cases, possibly from other large databases due to the rarity of this malignancy.

There is a paucity of literature comparing the outcomes of LC and COC in iGBC. In benign disease, COC has been associated with surgical site infections (SSI) [23]. A comparison of SSI rates between LC and COC iGBC cases could not be conducted due to a small sample size. Although this study found COC cases for iGBC to have significantly higher rates of postoperative sepsis and reoperation, COC for benign disease has not been associated with reoperation [24]. A possible confounder for this finding is that reoperations may have been the definitive treatment for the malignancy, a detail the database used could not distinguish. COC cases for our iGBC population had significantly longer hospital stays compared to non-converted cases. Similar findings were noted for surgeries with benign indications [24]. A prolonged recovery for these patients may delay, and possibly limit, additional oncologic-specific treatment post-surgery. Whether a more complicated recovery results in worse long-term oncologic outcomes in these patients warrants further study.

By using a large database, we were able to analyze operative patterns of a rare malignancy specifically when presenting in an incidental fashion. Our rate of incidental malignancy was similar to the literature and thus highlights the rarity of this cancer. Limitations to this study include a retrospective design, however, the focus on incidental presentation required our study to be undertaken in this fashion. Studies analyzing incidental malignancies are not designed prospectively, in which conditions are controlled. Another limitation was the low number of COC iGBC patients, which limited further analyses. 

The inability to review intraoperative records and operative pathology reports also limits this study. Accessing these reports may have helped address the confounders of COC due to a suspicion of malignancy and reoperation due to final diagnosis, respectively. Inherent to database investigations is the susceptibility to coding errors, biases, and variable data quality. Identification of COC cases required dual-labeling of procedures (laparoscopic and open). This was necessary to make use of this large database which addresses preoperative factors and surgical outcomes, both necessary to the primary and secondary aims of this study. A lack of insight into the intraoperative decision-making by the operating surgeons is another limitation. The ACS NSQIP database is not an oncologic database and therefore limits the findings to broad observations, without insight into long-term outcomes.

## Conclusions

The reported risk factors for COC in benign disease are not shared by those with iGBC. This study highlights the difficulty in predicting a COC for patients with undiagnosed malignancy. This prediction is challenging when an open surgical approach overlaps with definitive treatment at times. Also identified is the increased morbidity in the recovery of these patients when compared to benign disease. The increased postoperative complications noted with iGBC COC cases may lead to delays in adjuvant therapies needed for a malignant diagnosis.
